# Blocking TRAIL-DR5 signaling pathway with soluble death receptor 5 fusion protein mitigates radiation-induced injury

**DOI:** 10.3389/fphar.2023.1171293

**Published:** 2023-05-18

**Authors:** Danyang Zhao, Lei Yang, Peng Han, Haihui Zhang, Fanjun Wang, Zhiyun Meng, Hui Gan, Zhuona Wu, Wenzhong Sun, Chuan Chen, Guifang Dou, Ruolan Gu

**Affiliations:** ^1^ School of Life Sciences, Hebei University, Baoding, China; ^2^ Beijing Institute of Radiation Medicine, Beijing, China

**Keywords:** DR5, TRAIL, ARS, apoptosis antagonist, radiation damage effect

## Abstract

The increasing application of nuclear technology, the high fatality of acute radiation syndrome (ARS) and its complex mechanism make ARS a global difficulty that requires urgent attention. Here we reported that the death receptor 5 (DR5), as well as its ligand tumor necrosis factor-related apoptosis-inducing ligand (TRAIL), were both significantly upregulated after irradiation in mice with 6 Gy γ-ray single radiation. And by intravenously administrated with soluble DR5 fusion protein (sDR5-Fc), the competitive antagonist of DR5, the excessive apoptosis in the radiation-sensitive tissues such as spleen and thymus were significantly inhibited and the radiation-induced damage of spleen and thymus were mitigated, while the expression of apoptosis-inhibiting proteins such as Bcl-2 was also significantly upregulated. The biochemical indicators such as serum ALP, AST, ALT, TBIL, K, and Cl levels that affected by radiation, were improved by sDR5-Fc administration. sDR5-Fc can also regulate the number of immune cells and reduce blood cell death. For *in vitro* studies, it had been found that sDR5-Fc effectively inhibited apoptosis of human small intestinal mucosal epithelial cells and IEC-6 cells using flow cytometry. Finally, survival studies showed that mice administrated with sDR5-Fc after 9 Gy γ-ray single whole body radiation effectively increased the 30-day survival and was in a significant dose-dependent manner. Overall, the findings revealed that DR5/TRAIL-mediated apoptosis pathway had played important roles in the injury of ARS mice, and DR5 probably be a potential target for ARS therapeutics. And the DR5 apoptosis antagonist, sDR5 fusion protein, probably is a promising anti-ARS drug candidate which deserves further investigation.

## 1 Introduction

With the increasing use of nuclear technology in the military, industrial, and medical fields, the risk of accidental radiation exposure is also increasing. Acute radiation syndrome (ARS) is characterized by a series of signs after a whole or partial-body irradiation of sparsely ionizing, deeply penetrating radiation at high and intense doses (Singh and Seed, 2021). Many factors, such as the total body exposure, inhomogeneity of dose exposure, type of particles, absorbed dose, and dose rate, affect the time course and severity of clinical manifestations (Flynn and Goans, 2006; Macia et al., 2011; Hu, 2016). ARS is a comprehensive response of multiple organ failure which shows corresponding injury symptoms and signs caused by radiation, especially for the radiation sensitive organs such as spleen, thymus (Ohyama et al., 1985), bone marrow and gastrointestinal epithelium et al. Although it has been generally accepted that the biological effects of ionizing radiation start at the cellular level and the radiation damage effect is closely related to the excessive apoptosis (Low et al., 2006) of different tissue functional cells, and it has been studied that several targets were activated in the radiation-induced apoptosis such as p53, p38, ceramide, free radicals, et al. (Midgley et al., 1995; Takahashi et al., 2001; Alvarez et al., 2006), the complex signaling pathway network underlying radiation-induced apoptosis remains much unclear.

**FIGURE 1:**
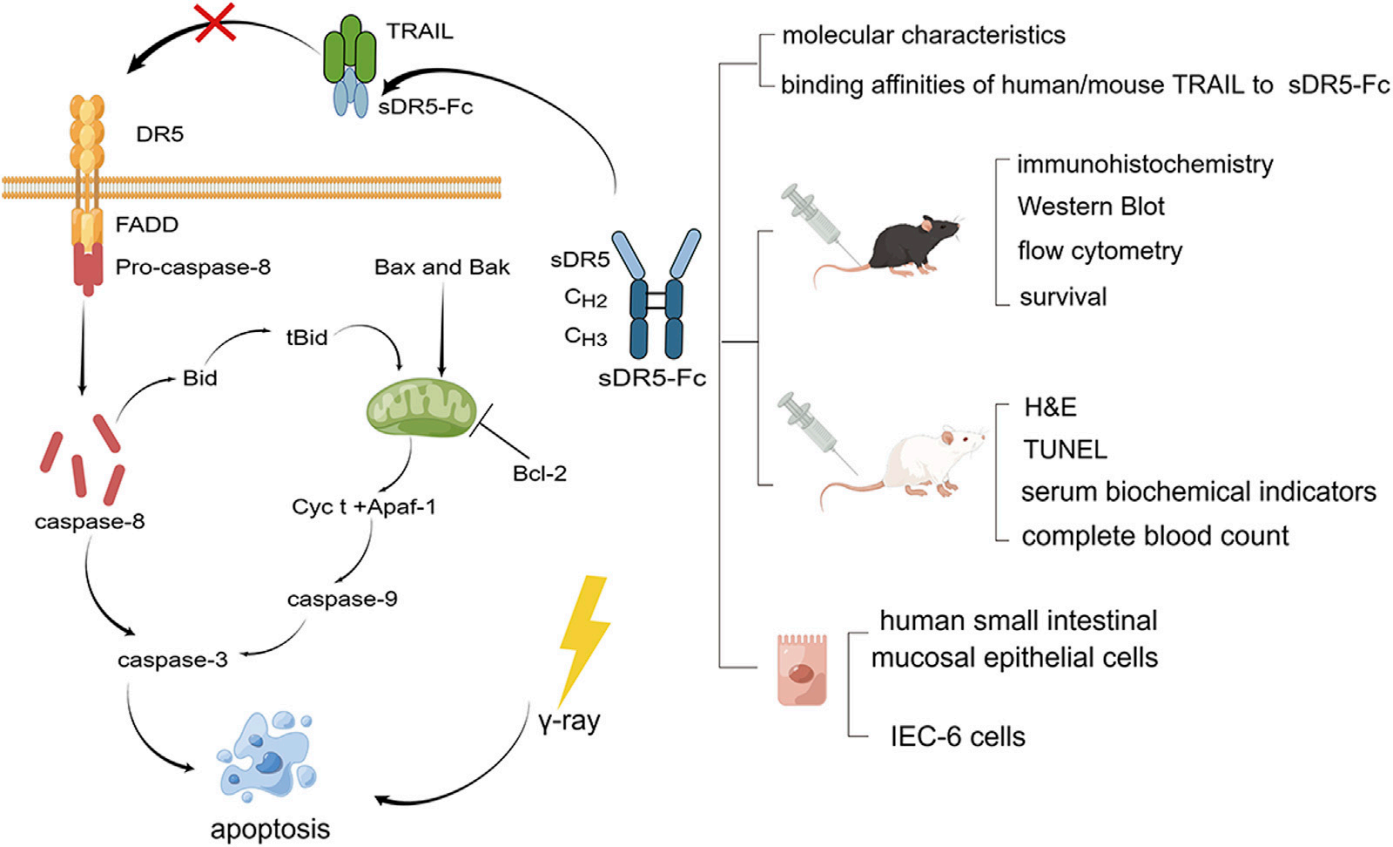
Graphical model of the study. TRAIL-DR5 signaling pathway and experimental technical route (By Figdraw).

Tumor necrosis factor (TNF)-related apoptosis-inducing ligand (TRAIL), a newly discovered member of the TNF family (Jeremias et al., 1998; Kayagaki et al., 1999), is expressed in many normal tissues including the spleen, thymus, lung, prostate and on the surface of T cells, B cells, macrophages, and natural killer cells (Wang and El-Deiry, 2003; Dadey et al., 2021). Because its high tumor specificity compared to other TNF family members, recombinant TRAIL, TRAIL receptor agonists and other therapeutic agents had been studied for cancer therapies by activating TRAIL pathway to induce tumor-selective apoptosis (Singh et al., 2021; von Karstedt et al., 2017; Yuan et al., 2018). DR5 is one of the TRAIL’s receptors with the highest affinity which can induce apoptosis when bound to TRAIL (Holoch and Griffith, 2009). It had been reported that DR5 was significantly upregulated under such abnormal conditions as virus and tumor, which was the reason why TRAIL agonists was investigated to treat cancer (Wu et al., 1999; Koliaki and Katsilambros, 2022). The native soluble DR5 (sDR5), which only contains the extracellular domain and lacks the transmembrane and intracellular regions and keeps the ability to bind to TRAIL, serves as an antagonist of DR5 to block TRAIL-DR5 pathway and inhibit apoptosis. sDR5-Fc fusion protein, by recombinant expression with human sDR5 and the Fc domain of human immunoglobulin G1, is more stable than sDR5 with a long half-life *in vivo* (Song et al., 2000; Peng et al., 2022). In previous studies, sDR5-Fc can effectively blocked TRAIL-induced apoptosis, inflammation and had good therapeutic effect on ischemia and heart-reperfusion (Wang et al., 2020), alleviate liver injury, inflammation, significantly reduce hepatocyte apoptosis and protect liver function (Chen et al., 2019; Chen et al., 2020), as well as reduce inflammation induced by SARS-CoV-2 (Peng et al., 2022). However, although many studies had focused on the possible therapeutic effects of TRAIL and its receptor DR5 in related diseases such as cancer and virus or inflammation-related disease, the role of TRAIL-DR5 pathway in ARS is unclear. The purpose of this study was to investigate the contribution of the TRAIL-DR5 signaling pathway to ARS caused by γ-ray in mice and the potential of sDR5-Fc to target this pathway for the treatment of ARS (Figure 1).

## 2 Experimental section

### 2.1 Materials and equipment

The human TRAIL, mouse TRAIL and human DR5 proteins were purchased from Sino Biological Inc. (10409-HNAE, 50166-M07E, and 10465-H02H, Beijing, China) and sDR5-Fc was provided by Amshenn Inc. Biotinylation Kit was obtained from Bomeida (G-MM-IGT, China). The primary antibodies used were TRAIL (ab231265, abcam), DR5 (ab8416, abcam), caspase8 (ab25901, abcam), Bcl-2 (ab196495, abcam), and β-actin (C1313, Applygen Techologies Inc.). 3,3′-diaminobenzidine (DAB) and counterstained with hematoxylin were supplied by Cloud-Clone (Wuhan, China). RIPA buffer, protease inhibitor cocktail, high-sensitivity ECL and Cell Counting Kit-8 were purchased from Applygen Techologies Inc. (Beijing, China). The bicinchoninic acid assay kit was from Thermo (MA, United States) and polyvinylidene difluoride (PVDF) membranes was from Immobilon^®^-PSQ (MA, United States). FITC Annexin V Apoptosis Detection Kit I was purchased from BD (New Jersey, United States). TUNEL Apoptosis Detection Kit and proteinase K were from Servicebio (Wuhan, China). Amifostine was obtained from MedChemExpress (New Jersey, United States).

Seven-week-old C57BL/6J male mice (19–21 g) and six-week-old Sprague-Dawley (SD) male rats (190–210 g) were purchased from the Keyu Animal Breeding Center (Beijing, China). All experimental procedures involving animals were approved by the Institution of Animal Care and Use Committee, Academy of Military Medical Science (IACUC-DWZX-2020-503, Beijing, China).

Human small intestinal mucosal epithelial cells (CP-H039, Procell, China) were cultured in a complete medium for human small intestinal mucosal epithelial cells (CM-H039, Procell, China) and Intestinal Epithelioid Cell line No. 6 (IEC-6 cells) were cultured in Dulbecco’s Modified Eagle Medium (812619, gibco, United States) with 10% fetal bovine serum (FBS) (SV30208.02, cytiva HyClone, United States)and 1% Penicillin Streptomycin (15140-122, gibco, United States) and at 37°C under 5% CO_2_ at atmosphere.

We used an Agilent 1200 HPLC system (Agilent Technologies, Santa Clara, United States) for size exclusion chromatography (SEC) coupled with a DAWN (Wyatt, Santa Barbara, United States) MALLS and an Optilab (Wyatt, Santa Barbara, United States) refractive index detector (RID) for molecular weight and purity determination. We used a TSKgel G4000PWXL (7.8 mm I.D. × 30 cm, TOSOH, Japan) as chromatographic column. The affinity to TRAIL of sDR5-Fc and DR5 and the kinetic analysis *in vitro* were conducted and compared using Octet^®^R2 Protein Analysis System (Sartorius, Germany) equipped with Streptavidin (SA) Biosensors (Sartorius, Germany). Images were acquired under an upright optical microscope (CK31, Olympus, Japan) using the MSHOT imaging system (TVO.63XC-MO, China), scanned using a digital full scan instrument (PANNORAMIC SCAN, 3D HISTECH). Blots were exposed using the Tanon (Shanghai, China). Flow cytometry was performed using a flow cytometer from BD (New Jersey, United States). Images of H&E staining were acquired under an upright optical microscope (Eclipse E100, Nikon, Japan). TUNEL assay were visualized and photographed using the Orth-Fluorescent Microscopy (ECLIPSE C1, Nikon, Japan), Imaging system (DS-U3, Nikon, Japan), Digital Slide Scanner (PANNORAMIC DESK/MIDI/250/1000, 3DHISTECH, Hungary). Serum biochemical indicators was detected by Hitachi (Tokyo, Japan). A cell counter (Mindray, Shenzhen, China) was used to measure complete blood count.

### 2.2 sDR5-Fc molecular weight and purity determination

A TSKgel G4000PW_XL_ chromatographic column was placed in an column oven maintained at 25°C. The mobile phase was 100 mM NaCl solution at the flow rate of 0.5 mL/min. sDR5-Fc was filtered through 22 mm diameter syringe filter after dilution. Injection volume was 100 μL of sDR5-Fc, and the constant mobile phase was eluted for 40 min. Data collection and processing were performed using the ASTRA software, Version 8.

### 2.3 TRAIL affinity analysis

The TRAIL protein was diluted in 1X phosphate-buffered saline (PBS) and Biotin-TRAIL was obtained through the desalination column after adding a 10 mM biotinylation reagent and performed at 25°C for 30 min. Subsequently, 10 μg/mL human Biotin-TRAIL and 20 μg/mL mouse Biotin-TRAIL were diluted in PBS/Tween (0.02% Tween, 1x PBS). Serially diluted human DR5 protein (Human DR5 7.81, 15.6, 31.3, 62.5, 125, and 250 nM) and sDR5-Fc (sDR5-Fc 7.81, 15.6, 31.3, 62.5, 125, and 250 nM) were added to a 96-well plate. Briefly, SA Biosensors were activated using freshly mixed PBST, with PBST serving as a running buffer. The human TRAIL was associated with human DR5 and sDR5-Fc with an association time of 120 s, and the buffer was maintained for 180 s for dissociation. An association time of 180 s was required when mouse TRAIL was associated with sDR5-Fc. K_D_ values were calculated using the Octet BLI Analysis software version 12.2 with a 1:1 binding model.

### 2.4 Animals

The mice and rats were housed in the Laboratory Animal Center barrier feeding system at the Academy of Military Medicine Sciences. Animals were kept in standard individually ventilated cages under standardized conditions (12-h light/dark cycle, 18°C–25°C, 50%–60% humidity) with food and water provided throughout the experiments.

A total of 147 male C57BL/6J were required for the experiment. 12 C57BL/6J mice were divided into control group, IR 24, 48, and 72 h groups, IR groups were radiated with 6 Gy γ-ray radiation through whole-body exposure for immunohistochemistry (*n* = 4). 9 C57BL/6J mice were divided into control group, model group, IR + 15 mg/kg sDR5-Fc groups for Western blotting. Model and administration groups were radiated with 6 Gy γ-ray radiation through whole-body exposure and administered with 15 mg/kg sDR5-Fc or normal saline via tail vein (*n* = 3). 30 C57BL/6J mice were divided into control group, model group, IR + 10 mg/kg sDR5-Fc groups for flow cytometry. Model and administration groups were radiated with 4 Gy γ-ray for apoptosis of the thymus. After 4 Gy γ-radiation, mediated groups mice were administered with 10 mg/kg sDR5-Fc via tail vein, control and model groups mice were treated with normal saline via tail vein (*n* = 6). 90 C57BL/6J mice were divided into control group, model group, positive drug group, IR + 5, 10, and 20 mg/kg sDR5-Fc groups for survival after 9 Gy γ-radiation (*n* = 15).

A total of 60 male SD rats weighed 190–210 g. SD rats were divided into control group, model group, 5, 10, and 15 mg/kg sDR5-Fc groups. SD rats were treated with 6 Gy γ-ray radiation through the whole-body exposure and administered with 5, 10, and 15 mg/kg sDR5-Fc via tail vein, control and model groups mice were treated with normal saline via tail vein for Hematoxylin-eosin (H&E) staining (*n* = 3), Terminal deoxynucleotidyl transferase dUTP nick end labeling (TUNEL) assay (*n* = 3), detection of serum biochemical indicators and complete blood count (*n* = 6).

### 2.5 The role of TRAIL-DR5 pathway in radiation injury

#### 2.5.1 Immunohistochemistry

Spleen and thymus tissues were fixed in 4% paraformaldehyde, embedded with paraffin, and sectioned. The rehydrated tissue sections were placed in a repair box filled with EDTA antigen repair buffer for antigen repair. The endogenous peroxidase activity was blocked using 3% H_2_O_2_ for 15 min at room temperature in the dark. After washing the sections thrice in PBS, tissues were blocked using 5% bovine serum albumin for 30 min at room temperature. The sections were incubated with TRAIL and DR5 antibodies at 4°C overnight, then incubated with horseradish peroxidase (HRP)- conjugated secondary antibodies at 37°C for 50 min. Histochemical development was visualized using 3,3′-diaminobenzidine (DAB) and counterstained with hematoxylin. Images were acquired under an upright optical microscope using the MSHOT imaging system, scanned using a digital full scan instrument and captured in the field of view using the Image-Pro Plus 6.0 software by a 200× standard ruler.

#### 2.5.2 Western blotting

RIPA buffer containing a protease inhibitor cocktail was added to the spleen samples. The protein mixture concentration was quantified using the bicinchoninic acid assay kit. Equal total protein amounts from different samples were separated by SDS-PAGE at 150 v for 1 h and then transferred to 0.2 μm PVDF membranes at 200 mA for 1 h. The membranes were blocked with 5% skim milk powder in Tris-buffered saline Tween (TBST) for 2 h at room temperature, then incubated with primary antibody at 4°C overnight. Before and after being treated with HRP-conjugated secondary antibody for 1 h at room temperature, the membranes were washed in TBST three times for 10 min each. Blots were visualized using high-sensitivity ECL, exposed using the Tanon and analyzed with ImageJ software.

#### 2.5.3 Flow cytometry

Mice thymuses were dissociated into single cells and harvested via centrifugation at 300 × g for 5 min. The cells were washed with PBS and labeled using the FITC Annexin V Apoptosis Detection Kit I. Subsequently, flow cytometry was performed using a flow cytometer.

### 2.6 Radiation protection efficacy of sDR5-Fc

#### 2.6.1 H&E staining

To explore the degree of tissue damage, we performed H&E staining. After removing the embedding agent from the sections, they were rehydrated and put into hematoxylin dyeing solution for 10 min and eosin solution for 10 min. Images were acquired under an upright optical microscope using Imaging System.

#### 2.6.2 TUNEL assay

The levels of apoptosis were detected using TUNEL Apoptosis Detection Kit. The rehydrated sections were digested with proteinase K at 37°C for 20 min and blocked with 3% H_2_O_2_ at room temperature for 20 min in the dark. Appropriate amounts of TDT enzyme, dUTP and buffer were collected using the TUNEL kit according to the number of sections and tissue size and mixed at a ratio of 1:5:50. The reagent streptavidin-HRP and Tris-buffered saline/Tween were mixed at a ratio of 1:200 and added to cover the tissue. The sections were incubated at 37°C for 30 min. The tissue was added with freshly prepared DAB chromogenic reagent to mark and counterstained in the nucleus with hematoxylin staining solution. The samples were visualized and photographed using the Orth-Fluorescent Microscopy, Imaging system, Digital Slide Scanner and analyzed using the 3DHISTECH CaseViewer2.4 and Halo v3.0.311.314 software.

#### 2.6.3 Detection of serum biochemical indicators

Serum was collected from rats administered sDR5-Fc or saline after 6 Gy γ-ray radiation and centrifuged at 3,000 r/min. Serum levels of alanine aminotransferase (ALT), aspartate aminotransferase (AST), alkaline phosphatase (ALP), total bilirubin (TBIL), potassium (K), and chlorine (Cl) were measured using a biochemistry analyzer.

#### 2.6.4 Detection of complete blood count

Whole blood was obtained from rats through the abdominal aorta and placed in EDTA k2 centrifuge tubes. After slight mixing, the cells were counted using a cell counter.

### 2.7 Survival

We treated C57BL/6J mice with 9 Gy ^60^Co γ-ray radiation at a dose rate of 53.94 Gy/min in the distance of 3 m from the irradiation source to the mice for survival rate and weight investigation for 1 month. After 9 Gy γ-ray radiation, mediated groups mice were administered with 5, 10, and 20 mg/kg sDR5-Fc via tail vein, control and model groups mice were treated with normal saline via tail vein (*n* = 15). Positive drug group mice were peritoneal injection with Amifostine 0.5 h before irradiation. The survival and weight of mice were monitored for 30 days.

### 2.8 *In vitro* cell experiments

#### 2.8.1 CCK-8 assay

Human small intestinal mucosal epithelial cells and IEC-6 cells were cultured in 96-well plates for 24 h at a concentration of 5000 cells per microliter. Then cells were irradiated at 5, 10, and 15 Gy and detected after 24 h with Cell Counting Kit-8.

Cell viability=100%×(OD−testODbackground)/(OD−controlODbackground) (1)

#### 2.8.2 Flow cytometry

Human small intestinal mucosal epithelial cells and IEC-6 cells were treated with 15 Gy γ-ray radiation and administered with or without 50 μg/mL sDR5-Fc. The cells were labeled with FITC Annexin V Apoptosis Detection Kit I.

### 2.9 Data and statistical analysis

All data are expressed as the mean ± standard error of mean (SEM). All analyses were performed using GraphPad Prism 8 (GraphPad Software, San Diego, CA). Differences between the experimental groups were assessed using one-way ANOVA or two-way ANOVA. *p ≤ 0.05 was considered statistically significant. “ns” was used to indicate a difference that was not significant.

## 3 Results and discussion

### 3.1 Characterization of the sDR5-Fc fusion protein

**FIGURE 2:**
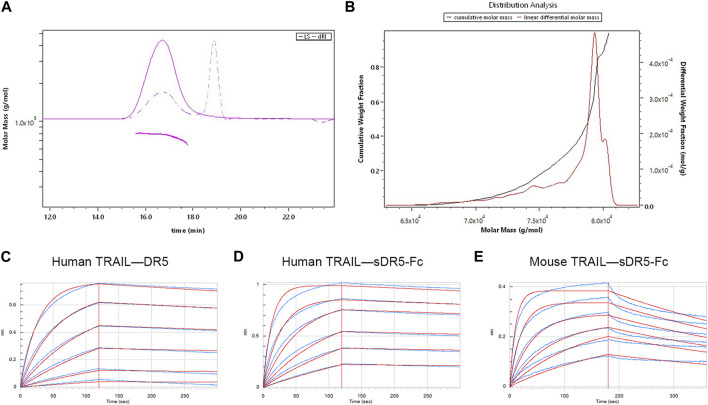
Characterization of the sDR5-Fc fusion protein. **(A)** Molecular mass of sDR5-Fc fusion protein. **(B)** Distribution analysis of different molar mass sDR5-Fc **(C,D)** Kinetic analysis of human TRAIL binding to human DR5 protein and sDR5-Fc. **(E)** Kinetics analyses of mouse TRAIL binding to sDR5-Fc.

We used SEC-MALLS to analyzed the molecular characteristics and purity of sDR5-Fc (Figures 2A, B; Table 1). The Mn and Mw of sDR5-Fc were 77.5 and 77.6 kDa. Polydispersity (Mw/Mn) was 1.001 and mass recovery was close to 100%. The results showed that the molecular weight distribution of sDR5-Fc was concentrated and the sample purity was close to 100%.

**TABLE 1:** Molecular characteristics of sDR5-Fc.

Sample	Mn (kDa)	Mw (kDa)	Mp (kDa)	Polydispersity (Mw/Mn)	Mass recovery (%)
sDR5-Fc	77.5	77.6	78.8	1.001	100.2

The binding affinities of human/mouse TRAIL for human DR5 and sDR5-Fc were determined by the Octet^®^R2 Protein Analysis System and the raw data (blue lines) were fitted to a 1:1 binding model (red lines) (Figures 2C–E). K_D_ values for the binding affinity between human TRAIL (10 μg/mL) and human DR5 and between human TRAIL and sDR5-Fc were 2.75E-09M and 9.68E-10M, respectively (up to 250 nM) (Figure 2C; Figure 2D; Table 2). Subsequently, we characterized the affinity of mouse TRAIL (20 μg/mL) binding to sDR5-Fc (up to 250 nM), and the KD value was found to be 5.71E-09M (Figure 2E) The binding affinity of human TRAIL for sDR5-Fc was significantly stronger than that of mouse TRAIL for sDR5-Fc. We first investigated the binding affinities of human/mouse TRAIL for sDR5-Fc and DR5 respectively, and found that sDR5-Fc had strong affinity with TRAIL *in vitro* and could competitively antagonize DR5 to bind with TRAIL.

**TABLE 2:** The affinity and kinetics data of the binding of TRAIL proteins species to sDR5-Fc.

Sample	Loading sample	K_D_(M)	k_d_ (1/s)	*R* ^2^
Human DR5	Human TRAIL	2.75E-09	4.01E-04	0.9979
sDR5-Fc	Human TRAIL	9.68E-10	2.91E-04	0.9938
sDR5-Fc	Mouse TRAIL	5.71E-09	2.09E-03	0.9797

### 3.2 DR5 and TRAIL are upregulated in the mice after irradiation

**FIGURE 3:**
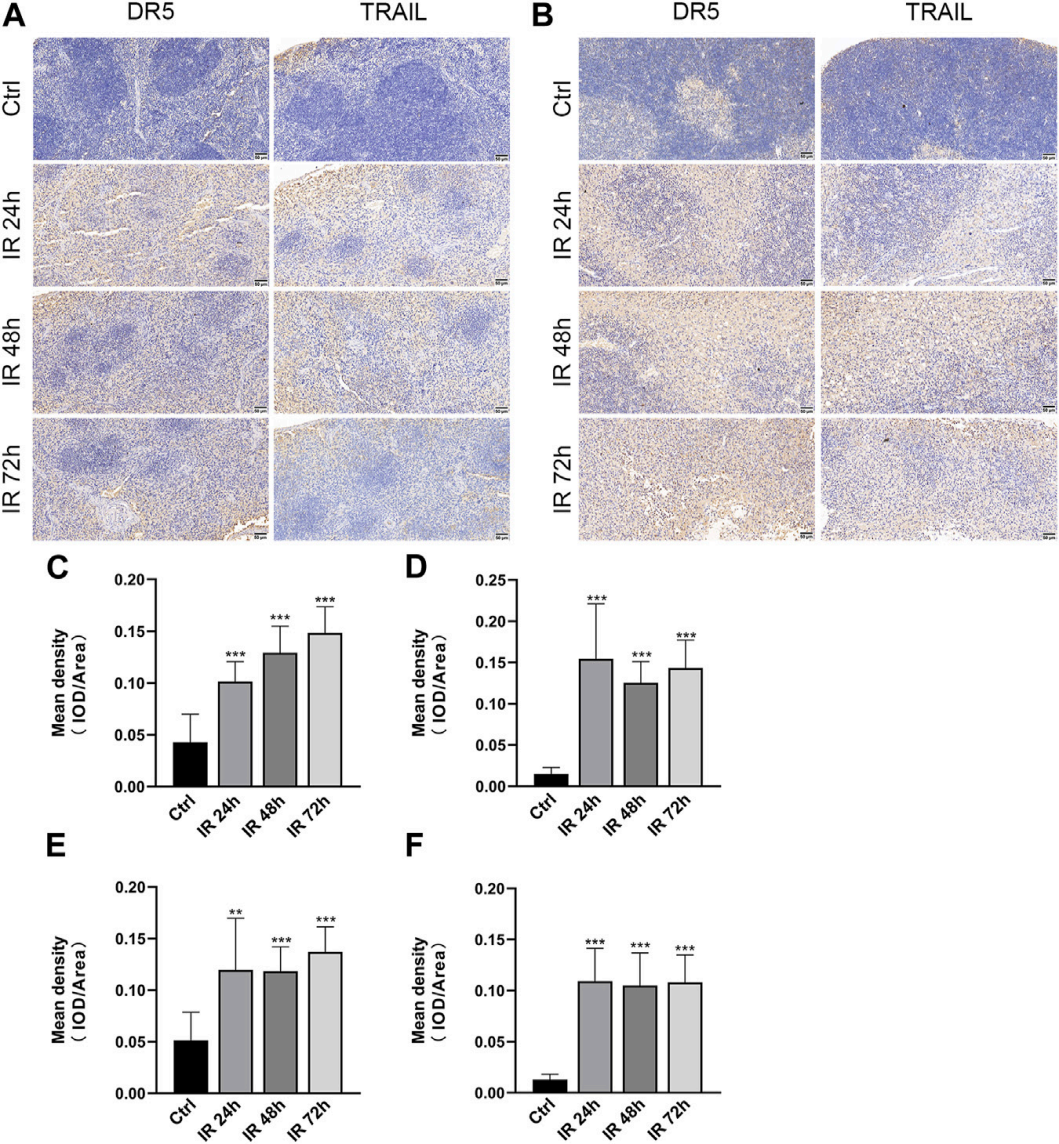
DR5 and TRAIL were upregulated in the spleen and thymus of mice after irradiation. **(A,B)** Immunohistochemistry staining of DR5 and TRAIL obtained from mice spleen**(A)** and thymus**(B)** after 24, 48, and 72 h of irradiation (*n* = 3). Scale bar, 50 μm. **(C–F)** Quantification of immunohistochemistry staining. IOD as percentages of the area of DR5 and TRAIL expressed in the spleen **(C,D)** and thymus **(E,F)**. Three fields of view are taken from each slice. Quantitative data are shown as means ± SEM. *p ≤ 0.05, **p ≤ 0.01, and ***p ≤ 0.001 as determined by one-way ANOVA.

To identify DR5 and TRAIL regulated by γ-irradiation in the spleen and thymus, we examined the expression of spleen and thymus protein via immunohistochemistry after 24, 48, and 72 h. We found that DR5 and TRAIL were upregulated in mice spleen and thymus after 24, 48, and 72 h of irradiation, compared with the control group (**p ≤ 0.01, ***p ≤ 0.001) (Figure 3).

Previously published studies had showed that γ-ray induce DR5 expression in spermatogonia (Coureuil et al., 2010) and DR5^−/−^ mice reduced radiation-induced apoptosis in spleen, thymus and colon compared to wild-type mice (Finnberg et al., 2005). However, it is not clear whether DR5 expression changes in normal tissues after radiation. Here we assessed the relationship between radiation and DR5/TRAIL expression using immunohistochemistry. And in our previous study, we have also found that the expression of DR5 protein and apoptosis cells in the thymus tissue of mice were significantly upregulated after irradiation by TUNEL/DR5 immunofluorescence double labeling, and the distribution and degree of apoptosis cells were basically consistent with the expression of DR5 protein (Cao et al., 2022). Both of which indicated that TRAIL-DR5-mediated apoptosis pathway had played important roles in γ-ray irradiation.

### 3.3 sDR5-Fc inhibits the DR5 and TRAIL-induced apoptosis signaling pathway

**FIGURE 4:**
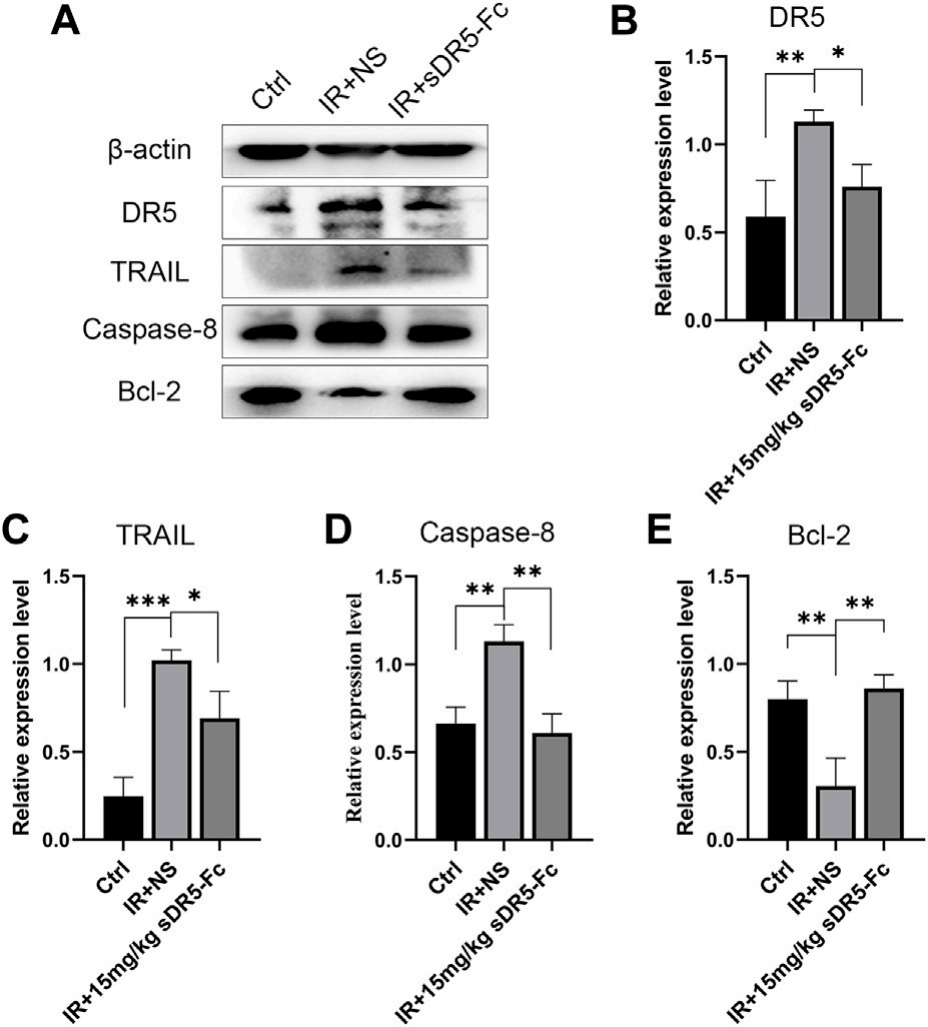
sDR5-Fc blocked DR5 and TRAIL-induced apoptosis signaling pathway. **(A)** Western blotting showing the expression levels of proteins in mice spleen after irradiation with 15 mg/kg sDR5-Fc **(B–E)** Relative protein levels of DR5 **(B)**, TRAIL **(C)**, Caspase-8 **(D)** and Bcl-2 **(E)**. Quantitative data are shown as means ± SEM. *p ≤ 0.05, **p ≤ 0.01, and ***p ≤ 0.001 as determined using one-way ANOVA.

To explore the blocking effect of sDR5-Fc on protein expression level, we used Western blotting to detect the expression changes in apoptotic pathway proteins in the spleen (Figure 4). After irradiation, the expression of DR5, TRAIL and Caspase-8 was upregulated and apoptosis-inhibiting protein Bcl-2 was downregulated. DR5, TRAIL, and Caspase-8 were downregulated, and Bcl-2 was upregulated in mice treated with 15 mg/kg sDR5-Fc after irradiation (*p ≤ 0.05, **p ≤ 0.01, and ***p ≤ 0.001). In order to further verify whether DR5 can be used as a potential target for ARS treatment, the DR5 antagonist sDR5-Fc were used. Then, *in vivo* animal studies had conducted and Western blotting revealed that injection of sDR5-Fc after irradiation efficiently blocked the TRAIL/DR5 apoptosis pathway.

However, for apoptosis studies, cleaved caspases measurement is the gold standard which can significantly reduce the possible interference due to the background or saturation issues of Western blot. In the present study there is a deficiency for not detecting cleaved caspases due to some insufficient conditions, and which should be supplemented in future study to further verify the role of TRAIL/DR5 apoptosis pathway in radiation damage.

### 3.4 sDR5-Fc attenuates radiation-induced tissue injury

**FIGURE 5:**
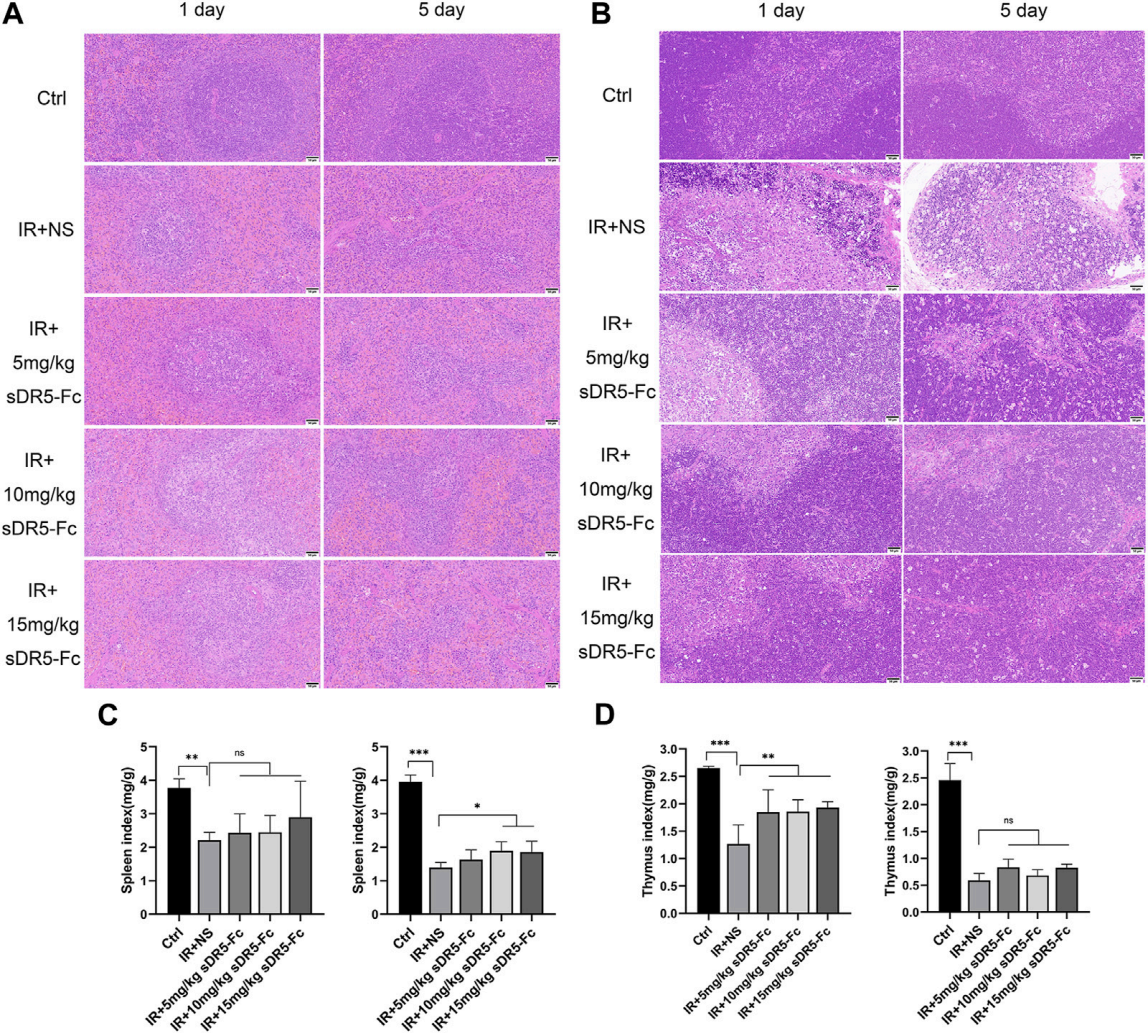
sDR5-Fc prevented tissue injury in rats after irradiation. **(A)** Spleen and **(B)** thymus injuries were determined using H&E staining after 6 Gy irradiation with or without sDR5-Fc on the first and fifth days (*n* = 3). Scale bars, 50 μm **(C,D)** The spleen **(C)** and thymus **(D)** indices were showed (*n* = 6 rats). Quantitative data are shown as means ± SEM. *p ≤ 0.05, **p ≤ 0.01, and ***p ≤ 0.001, as determined by one-way ANOVA.

We used H&E staining to examine the repair effect of sDR5-Fc on spleen and thymus injury after radiation. Rats were injected with sDR5-Fc after whole-body irradiation with 6 Gy γ-ray. After the radiation, the rat spleen capsule was thickened, the amount of white pulp decreased significantly, and the volume of residual white pulp became smaller. The marginal zone disappeared, the structure was distorted, and the number of fibrocytes increased slightly. A substantial decrease in the number of lymphocytes, mild proliferation of histiocytes in the red pulp, a slight increase in the spleen trabeculae were observed and small amount of neutrophil infiltration (Figure 5A). The thymus tissue atrophied and became smaller, with severe injury. The medulla structure was almost disrupted, and a few collagen fibers were proliferated. Many lymphocytes were necrotic, and the structure disrupted, exhibiting a starry sky appearance (Figure 5B). The spleen and thymus injuries were recovered in rats injected with sDR5-Fc after radiation. The spleen and thymus indices decreased significantly on the first and fifth days after irradiation (**p ≤ 0.01, ***p ≤ 0.001). The spleen index increased on the fifth day after irradiation with 10 and 15 mg/kg sDR5-Fc, compared with the group treated with the normal saline after receiving IR (*p ≤ 0.05) (Figure 5C). Thymus index increased on the first day of irradiation with 5, 10, and 15 mg/kg sDR5-Fc (**p ≤ 0.01) (Figure 5D). Because the immunomodulatory system is highly sensitive to irradiation, exposure to irradiation could cause damage to the spleen and thymus and lead to apoptosis (Cheng et al., 2018). Here the H&E staining and organ indices results had showed that sDR5-Fc could reduce radiation-induced spleen and thymus injury.

### 3.5 sDR5-Fc inhibits apoptosis induced by irradiation

**FIGURE 6:**
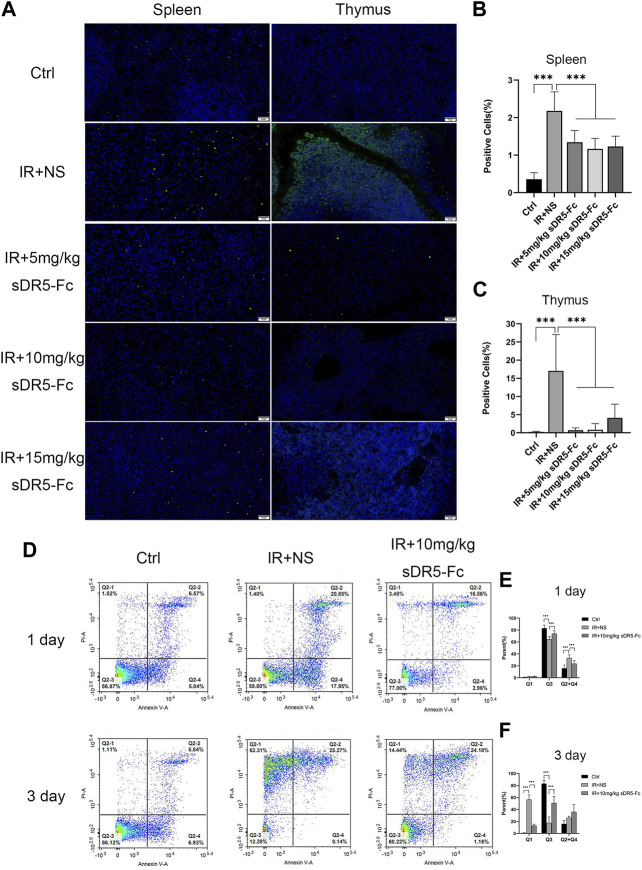
sDR5-Fc reduced cell apoptosis after irradiation. **(A)** TUNEL assay was used to evaluate apoptotic cells in the spleen and thymus after 24 h of 6 Gy irradiation treated with or without sDR5-Fc (*n* = 3). Scale bars, 50 μm. **(B,C)** Quantification of positive cells as percentages of the TUNEL assay in the spleen and thymus are shown in **(B)** and **(C)**. Three fields of view were taken from each slice. **(D)** Apoptotic cells were isolated from the thymus of mice after the first day and third day of 4 Gy irradiation with or without sDR5-Fc, as determined using flow cytometry assay (n = 6). **(E,F)** Quantification of the flow cytometry assay after the first day**(E)** and third day**(F)** of irradiation. Quantitative data are shown as means ± SEM. *p ≤ 0.05, **p ≤ 0.01, ***p ≤ 0.001 and ns = no significant, as determined using one-way ANOVA.

We used TUNEL assay to assess apoptosis of the spleen and thymus in rats. The number of apoptotic cells was increased significantly in the spleen and thymus after 24 h of irradiation. sDR5-Fc administration after irradiation reduced apoptosis by blocking TRAIL as determined by TUNEL (***p ≤ 0.001) (Figures 6A–C). Additionally, 10 mg/kg sDR5-Fc administered after 4 Gy irradiation significantly reduced the apoptosis of thymus cells on the first and third days after irradiation (***p ≤ 0.001) (Figures 6D–F).

### 3.6 Effects of sDR5-Fc on serum biochemical indicators and complete blood count

**FIGURE 7:**
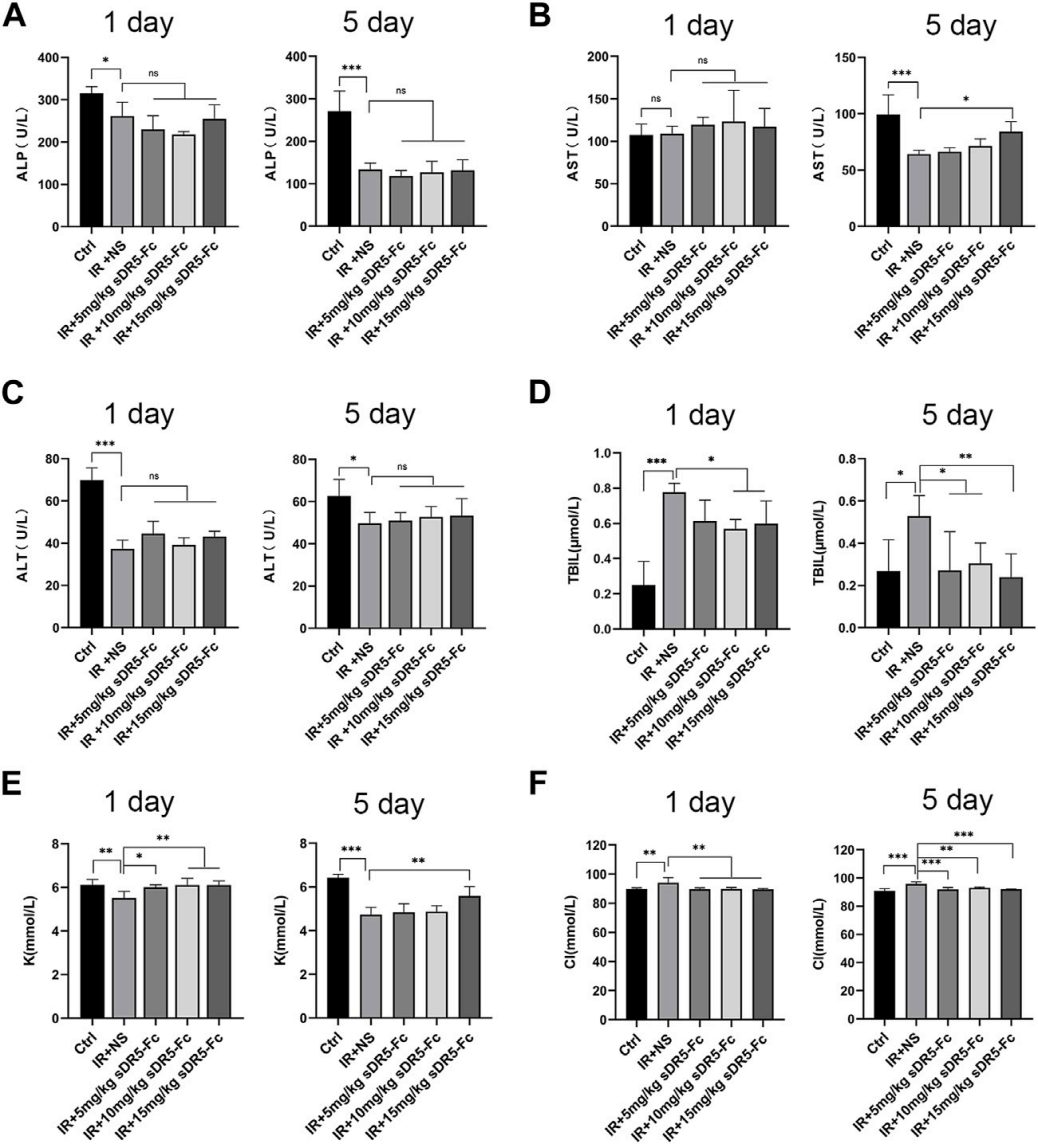
Effects of sDR5-Fc on serum biochemical indicators. Serum ALP **(A)**, AST **(B)**, ALT **(C)**, TBIL **(D)**, K **(E)** and Cl **(F)** levels were quantified after 6 Gy irradiation with or without sDR5-Fc treated on the first and fifth day (*n* = 6). Quantitative data are shown as means ± SEM. *p ≤ 0.05, **p ≤ 0.01, ***p ≤ 0.001, and ns = no significant as determined using one-way ANOVA.

We evaluated the serum biochemical indicators of rats after 6 Gy γ-ray irradiation and again after administering different concentration of sDR5-Fc (Figure 7). Serum ALP and ALT levels decreased after irradiation on the first and fifth day. Serum AST levels decreased after irradiation and increased after irradiation with 15 mg/kg sDR5-Fc on the fifth day (*p ≤ 0.05). Serum TBIL levels increased significantly after irradiation and decreased in rats injected with sDR5-Fc on the first and fifth day (*p ≤ 0.05, **p ≤ 0.01, ***p ≤ 0.001). Serum K levels decreased after irradiation and increased in rats injected with all doses of sDR5-Fc on the first day and 15 mg/kg sDR5-Fc on the fifth day (*p ≤ 0.05, **p ≤ 0.01, ***p ≤ 0.001). Serum Cl increased after irradiation and decreased in rats injected with all doses of sDR5-Fc on the first and fifth day (**p ≤ 0.01, ***p ≤ 0.001). Irradiation causes disorder of serum biochemical indicators. The decrease in serum biochemical indicators of liver function, such as ALT, AST, and ALP, might be attributed to a transitory reduction in the release of ALP to the enzymatic circulation by rapidly metabolizing cells and injury to the intestinal mucosa after irradiation (Abdel-Magied et al., 2018). In this study, sDR5-Fc administration increased serum AST level on the fifth day after irradiation. The increase of TBIL level caused by radiation exacerbates secondary complications of stress, inflammation, and infection (Khan and Poduval, 2012). In this study, sDR5-Fc inhibited the increase in TBIL level caused by 6 Gy γ-ray irradiation. Meanwhile, radiation exposure results in electrolyte disturbances, including a decrease in K and an increase in Cl levels. This might be attributed to alterations in renal homeostatic mechanisms and changes in cell membrane permeability (Nwokocha et al., 2012). And our results had showed that sDR5-Fc could regulate such radiation-induced electrolyte disturbances.

**FIGURE 8:**
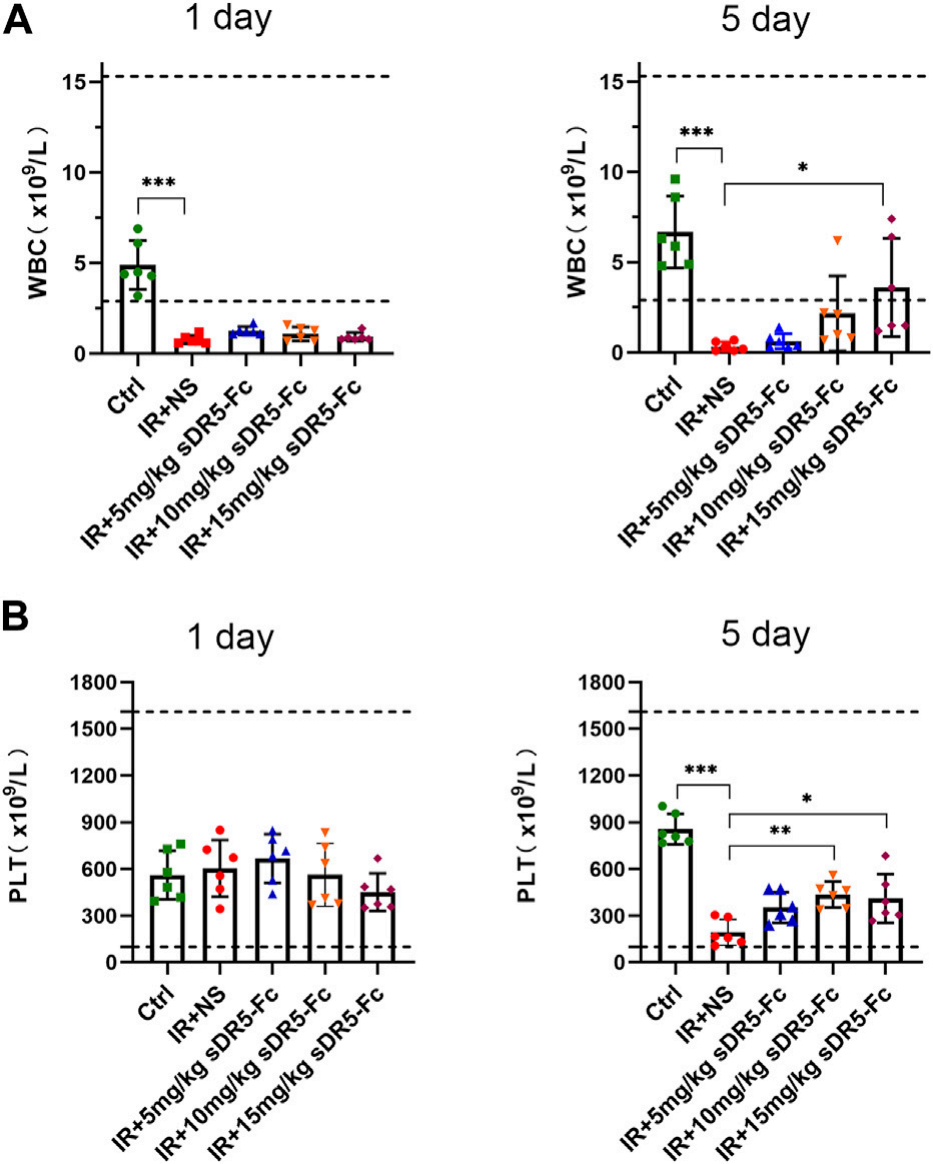
Effects of sDR5-Fc on complete blood count. WBC **(A)** and PLT **(B)** were quantified after 6 Gy irradiation with or without sDR5-Fc treated on the first and fifth day (*n* = 6). Quantitative data are shown as means ± SEM. *p ≤ 0.05, **p ≤ 0.01, ***p ≤ 0.001, and ns = no significant as determined using one-way ANOVA.

We counted the number of white blood cells and platelets in the whole blood cells of SD rats after irradiation (Figure 8). On the first and fifth day after irradiation, the number of white blood cells decreased sharply to below the normal range as compared with the control group. Compared with the model group, the number of white blood cells in the sDR5-Fc group showed an upward trend with the increase of dose on the fifth day after radiation. Compared with the control group, the number of platelets decreased sharply on the fifth day after radiation. Compared with the model group, the number of platelets in the sDR5-Fc treatment group increased. The difference between the 10 and 15 mg/kg sDR5-Fc administration group and the model group was statistically significant (*p ≤ 0.05, **p ≤ 0.01, ***p ≤ 0.001). It plays a role in regulating the number of immune cells and reducing blood cell death.

### 3.7 sDR5-Fc blocked radiation-induced apoptosis in intestinal cells

**FIGURE 9:**
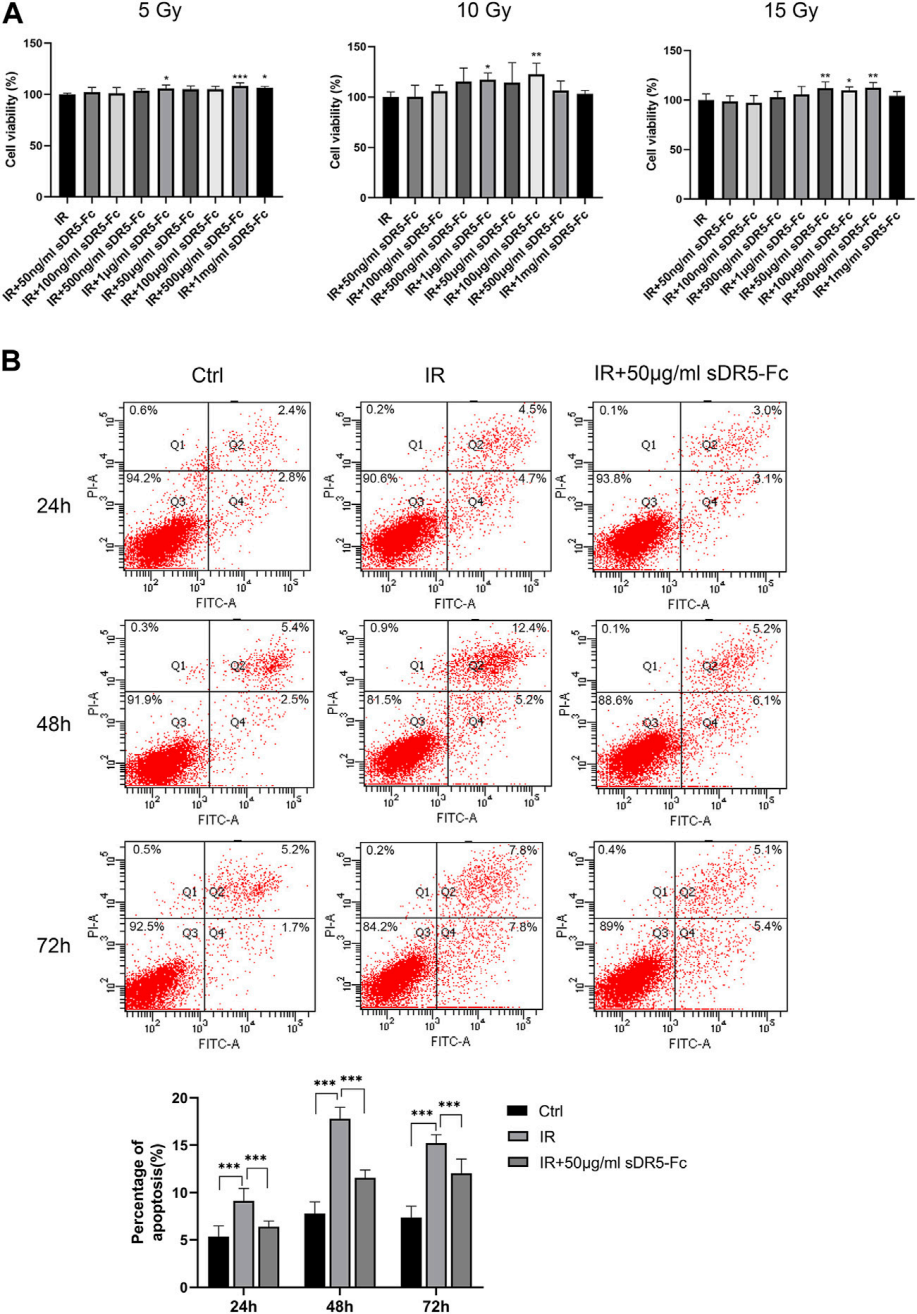
sDR5-Fc blocked apoptosis in human small intestinal mucosal epithelial cells after irradiation. **(A)** Viability of human small intestinal mucosal epithelial cells at different radiation and administration doses. **(B)** Apoptosis percentage in human small intestinal mucosal epithelial cells at 24, 48 and 72 h after irradiation with or without 50 μg/mL sDR5-Fc. Quantitative data are shown as means ± SEM. *p ≤ 0.05, **p ≤ 0.01, and ***p ≤ 0.001, as determined using one-way ANOVA and two-way ANOVA.

**FIGURE 10:**
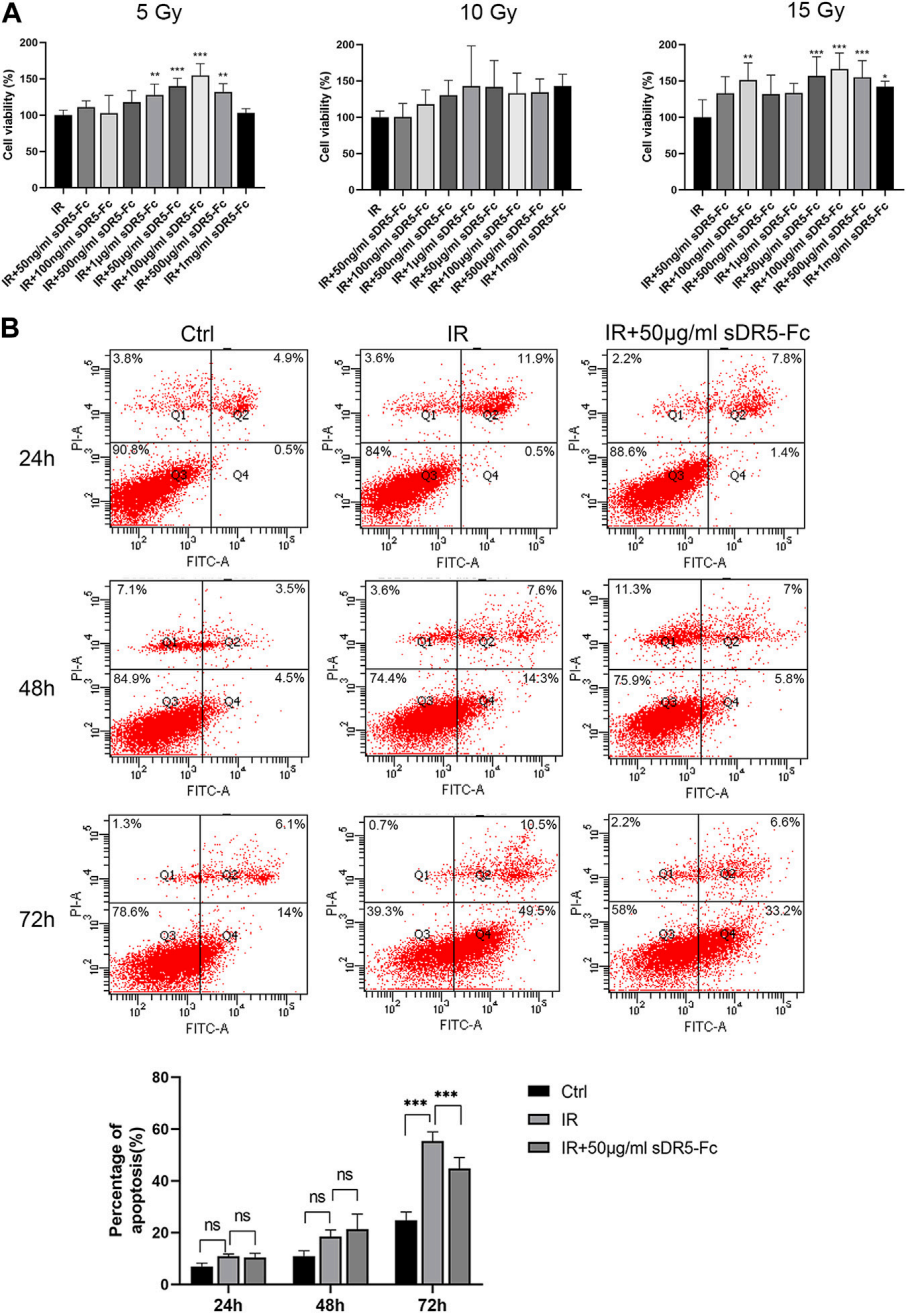
sDR5-Fc blocked apoptosis in IEC-6 cells after irradiation. **(A)** Viability of IEC-6 cells at different radiation and administration doses. **(B)** Apoptosis percentage in IEC-6 cells at 24, 48 and 72 h after irradiation with or without 50 μg/mL sDR5-Fc. Quantitative data are shown as means ± SEM. *p ≤ 0.05, **p ≤ 0.01, and ***p ≤ 0.001, as determined using one-way ANOVA and two-way ANOVA.

Colony formation assay and CCK-8 method are both used for detecting the cell proliferation activity after radiation injury. In order to quickly screen the optimum radiation dose and sDR5-fc administration dose, we used the CCK-8 assay to examine the cell viability of human small intestinal mucosal epithelial cells (Figure 9A) and IEC-6 cells (Figure 10A) at 5, 10, and 15 Gy radiation and various sDR5-Fc administration doses *in vitro*. 15 Gy was selected as radiation dose to establish cells injury model, and 50 μg/mL sDR5-Fc was selected as the administration dose. Flow cytometry revealed that sDR5-Fc decreased the percentage of apoptotic cells and successfully blocked radiation-induced apoptosis in human small mucosal intestinal epithelial cells after 24, 48, and 72 h (Figure 9B) and blocked IEC-6 cells (Figure 10B) after 72 h (***p ≤ 0.001). Among these three time points, the apoptosis of human small intestinal mucosal epithelial cells was the most evident and sDR5-Fc was the most effective in blocking apoptosis at 48 h after radiation. Radiation damages the intestinal mucosa and causes acute radiation enteritis (Singh and Seed, 2019; Fan et al., 2022). The intestinal mucosal barrier plays a crucial role in protecting against the invasion of foreign antigens, maintaining the stability of the internal environment, as well as the normal activities of the body (Turner, 2009). As the first cells to come in contact with antigens, intestinal mucosal epithelial cells play a key role in the initial stage of the mucosal immune response by determining its occurrence, nature, and intensity (Kayama et al., 2020). In this study, we found that sDR5-Fc could significantly reduce the radiation-induced apoptosis of human small intestinal mucosal epithelial cells and IEC-6 cells, which provided a basis and possibility of sDR5-Fc for the subsequent treatment of intestinal acute radiation syndrome.

### 3.8 sDR5-Fc can improve percent survival of irradiated mice

**FIGURE 11:**
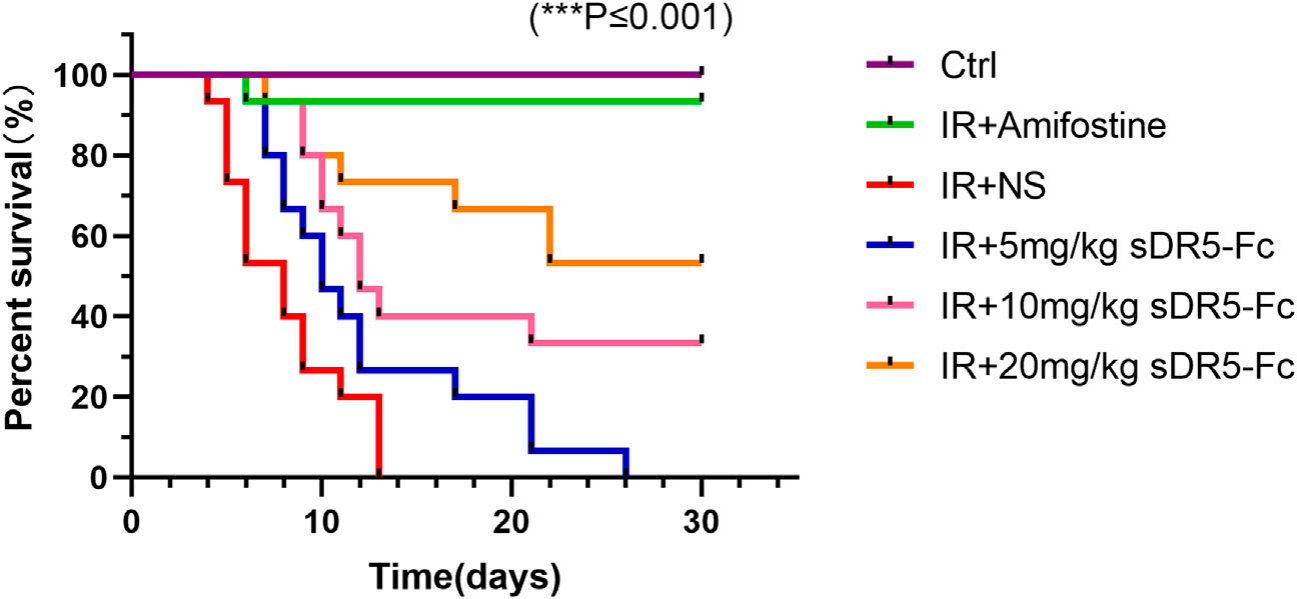
Percent survival of mice after 9 Gy irradiation and administration with sDR5-Fc. (*n* = 15). Quantitative data are shown as means ± SEM. *p ≤ 0.05, **p ≤ 0.01, and ***p ≤ 0.001 as determined by one-way ANOVA.

We observed the survival of the mice for 1 month after radiation (Figure 11). All mice in the model group died within 13 days after 9 Gy γ-ray radiation. The survival rate of mice in sDR5-Fc administration groups increased, and the survival rate of mice increased with the increase of administration dose. To further confirm the efficiency of sDR5-Fc on the survival of ARS mice after high doses of radiation, mice were irradiated with a single whole-body dose of 9 Gy γ-rays. The results had showed that amifostine had good effects on ARS (Ormsby et al., 2014), but it must be administered within 30 min before irradiation, the narrow prophylactic administration window obviously limited its application in the clinical application for radiation injury. Amifostine also has toxic side effects such as hypotension, vomiting, nausea, drowsiness, allergic rash, fever or shock. Our study demonstrated that sDR5-Fc could improve the survival rate of ARS mice by administration after radiation, which had a good post-radiation therapeutic effect and is of great value for the emergency treatment of radioactive accidents. For the determination of the complete therapeutic window of sDR5-Fc in the treatment of ARS, we will further combine the pharmacokinetic characteristics of sDR5-Fc *in vivo* to design the efficacy and safety evaluation of different time points and multiple doses after radiation.

## 4 Conclusion

In conclusion, our study suggested that DR5/TRAIL signaling pathway had played an important role in ARS and probably is a new therapeutic target for the treatment of ARS. sDR5-Fc could reduce radiation-induced excessive apoptosis and tissue injury by inhibiting TRAIL/DR5 pathway. Meanwhile, sDR5-Fc can regulate serum biochemistry and the number of complete blood cells, and improve the survival in severe acute radiation syndrome, which is a promising candidate for the treatment of ARS and deserved further evaluation.

## Data Availability

The original contributions presented in the study are included in the article/Supplementary Material, further inquiries can be directed to the corresponding authors.
